# Occupational Exposure to Diisocyanates in the European Union

**DOI:** 10.1093/annweh/wxab021

**Published:** 2021-04-23

**Authors:** Dag Rother, Urs Schlüter

**Affiliations:** Federal Institute for Occupational Safety and Health (BAuA), Division 4 - Hazardous Substances and Biological Agents, Unit 4.1 - Exposure Scenarios, Friedrich-Henkel-Weg 1, Dortmund, Germany; Federal Institute for Occupational Safety and Health (BAuA), Division 4 - Hazardous Substances and Biological Agents, Unit 4.1 - Exposure Scenarios, Friedrich-Henkel-Weg 1, Dortmund, Germany

**Keywords:** asthma, diisocyanates, occupational exposure, workplace exposure

## Abstract

**Objectives:**

Diisocyanates are a chemical group that are widely used at workplaces in many sectors. They are also potent skin- and respiratory sensitizers. Exposure to diisocyanates is a main cause of occupational asthma in the European Union. To reduce occupational exposure to diisocyanates and consequently the cases of diisocyanate-induced asthma, a restriction on diisocyanates was recently adopted under the REACH Regulation in the European Union.

**Methods:**

A comprehensive evaluation of the data on occupational exposure to the most important diisocyanates at workplaces was made and is reported here. The diisocyanates considered are methylene diphenyl diisocyanate (MDI), toluene diisocyanate (TDI), and hexamethylene diisocyanate (HDI), accounting for more than 95% of the market volume in the EU. The exposure assessment is based on data from Chemical Safety Reports (CSRs) of REACH Registration Dossiers, workplace air monitoring data from Germany, from the UK Health and Safety Executive (HSE), and literature data relevant for the EU, and the USA.

**Results:**

Occupational exposure to diisocyanates is particularly relevant in: (i) C.A.S.E. applications (Coatings, Adhesives, Sealants, Elastomers), (ii) production of polyurethanes (PUs) (e.g. slab-stock foam), (iii) handling of partly uncured PU products (e.g. cutting, demoulding, spray application of foam), and (iv) when diisocyanates/PUs are heated (e.g. hot lamination, foundry applications/casting forms). Ranking of the reported data on inhalation to diisocyanate exposure at workplaces (maximum values) leads to following order: (i) HDI and its oligomers in coatings, (ii) MDI in spray foam applications, (iii) TDI in manufacture of foam, (iv) TDI in manufacture of PUs and PU composite materials, (v) TDI in adhesives, (vi) MDI in adhesives, (vii) MDI in manufacture of PUs and PU composite materials, (viii) TDI in coatings, (ix) MDI in manufacture of foam, and (x) HDI in adhesives.

**What’s important about this paper**

Exposure to diisocyanates is a main cause of occupational asthma in the European Union. To reduce occupational exposure to diisocyanates and consequently the cases of diisocyanate-induced asthma, a restriction on products containing more than 0.1% by weight of diisocyanates was recently adopted under the REACH Regulation in the European Union. A comprehensive evaluation of the data on occupational exposure to the most important diisocyanates at workplaces was made in course of the restriction proposal.

## Introduction

Isocyanates are highly reactive compounds defined by the isocyanate group, R–N=C=O, where R can be an aliphatic, cycloaliphatic, or an aromatic group. Isocyanates undergo exothermic and usually very fast reactions with nucleophiles. The most common types of isocyanates in workplaces are diisocyanates and oligomers/polyisocyanates derived thereof, which act as cross-linking agents. Their predominant use (>90%) is in the manufacture of polyurethane plastic materials (PUs, also PURs) by reacting with polyols and/or other nucleophiles like polyamines. Depending on the choice of the diisocyanate species and the polynucleophiles a wide range of polymers with diverse properties can be realized (Engels *et al.*, 2013). Consequently, the use of diisocyanates at the workplace is just as diverse and widespread.

Typical diisocyanate-based products include:

• flexible PUs
• rigid PUs
• PU foams (rigid and flexible foam systems, e.g. mattresses)
• assembly foams (e.g. insulation panels)
• foundry cores (casting)
• coating materials (paints, lacquers, varnishes)
• adhesives and glues
• elastomers
• sealants
• prepolymers in chemical synthesis
• engineering plastics
• PU fibres

Notwithstanding their versatile material properties isocyanates are also potent respiratory sensitizers, making exposure to diisocyanates the main cause of occupational asthma in Germany and in the European Union. The number of new cases of occupational asthma resulting from exposure to diisocyanates in the EU is estimated to be more than 5000 per year. As this number is considered to be unacceptably high, Germany has prepared a restriction dossier for diisocyanates under REACH and submitted it to the European Chemicals Agency (ECHA). The restriction proposes to limit the use of diisocyanates to those workplaces where appropriate technical and organizational measures have been implemented and workers received a standardized training package to prepare them for appropriate risk management measures in a most efficient way and thus effectively lower/minimize exposures to diisocyanates and the associated risks.

In the course of the restriction dossier preparation, also a comprehensive evaluation of the current data on toxicology, epidemiology, and exposure to diisocyanates from various sources was made. Additionally, a socioeconomic impact assessment of the cost and benefits of the proposed restriction in comparison with two other risk management options and a baseline scenario (i.e. no further action) has been made. As part of the dossier preparation, an overview of the exposure situation in the EU was compiled of which the results are presented in this paper. Updates to the initial assessment were also added in the course of drafting the paper.

## Scope and method of the assessment

In the restriction dossier, overall 10 different types of diisocyanates are included in a non-exhaustive list of diisocyanates covered by the restriction proposal. However, there are large differences with regard to the market volumes and used quantities of different diisocyanate species. The most important diisocyanates in terms of quantities used are:

- methylene diphenyl diisocyanate (MDI),
- toluene diisocyanate (TDI), and
- hexamethylene diisocyanate (HDI),

which together account for more than 95% of the market volume (Falcke *et al*., 2017). Consequently, most of exposure monitoring data are on MDI, TDI, and HDI. For this reason, the exposure assessment in the restriction dossier was limited to the three diisocyanates with the highest market volume rather than a comprehensive list of all diisocyanates, where only very limited information on the exposure was found. Since most of the published data are based on the measurement of the diisocyanate monomers, a focus is put on them.

MDI and TDI are aromatic diisocyanates, i.e. the NCO groups are attached to an aromatic ring, leading to particularly highly reactive diisocyanate species due to mesomeric interactions. They react extremely fast with polyols under catalysed conditions to PUs. Depending on the chemical nature of the chemical building blocks (type of isocyanates and polyols), a wide range of PUs with tailored material properties can be realized (soft elastic to hard).

Aliphatic diisocyanates as HDI, i.e. where the NCO group is not directly attached to an aromatic ring, have lower reactivity, but also form more inert urethane bonds than aromatic diisocyanates. PU materials made thereof are very durable and exhibit higher ultraviolet stability as well as chemical and mechanical resistance compared with PUs based on aromatic diisocyanates. Aliphatic diisocyanates, with HDI being the most commonly used species, are generally regarded as speciality materials and account for less than 5% of the total diisocyanate consumption. The main applications are as hardeners for high quality surface coatings, where high performance is required, and as adhesives.

For the exposure assessment in the restriction dossier the following sources of information were evaluated and the assessment basically is based on these:

- Chemical Safety Reports (CSRs) of the Registration Dossiers [prepared by the Netherlands Organisation for Applied Scientific Research (TNO), where the exposure estimates are based on measurement data published by ISOPA (2012a,b, 2014a,b,c)] and on modelled data [Advanced REACH Tool (ART) v 1.0];
- Workplace measurement data (gathered from 2000 to 2011) from Germany published by the Institute for Occupational Safety and Health of the German Social Accident Insurance (IFA, 2010, 2012, 2013);
- Workplace air monitoring data from the UK Health and Safety Executive (HSE); and
- Selected and evaluated literature data relevant for the EU (this includes data since 2000 mainly from Belgium, Finland, the Netherlands, Poland, Sweden, Spain, the UK and the USA).

The literature research was done using public databases (Web of Science, Google Scholar, and PubMed) and standard search engines (such as Ecosia and Google). Selection of the literature was made according to timeliness (since 2000) and relevance for the situation in the EU, but not limited to the Member States of the European Union. The search strategy included different terms for diisocyanates (‘isocyanate’ OR ‘diisocyanate’ OR ‘MDI’ OR ‘TDI’ OR ‘HDI’) and exposure. The research was then further narrowed down to studies with a focus on workplaces or occupational settings [‘exposure’ AND (‘occupational’ OR ‘worker’ OR ‘workplace’)]. The remaining titles and abstracts were screened and about 200 matches were obtained in full text out of which the 58 quoted papers were selected.

The data from the CSRs presented here are based on the information published by ISOPA (2012a,b, 2014a,b,c) and no data from CSRs not publically available are presented for confidentiality reasons in this publication. However, the full dataset, as available in the registration dossiers (including confidential data) was evaluated for the preparation of the restriction dossier.

In the beginning of the exposure assessment, a request was made to the Institute for Occupational Safety and Health of the German Social Accident Insurance (IFA) in addition to the other member states of the European Union via ECHA for data on occupational exposure to diisocyanates. The IFA made exhaustive evaluations of workplace measurement data from Germany for MDI, TDI, and HDI by IFA (2010, 2012, 2013). These data were representative for more than 6 h of time of workplace exposure and documented in accordance to the measurement system of the German Social Accident Insurance Institutions for exposure assessment (MGU) (Gabriel *et al*., 2010). Grouping of the data was done according to industry groups as well as work area groups. However, for drafting the restriction dossier, the available information on occupational exposure was grouped differently, i.e. according to the information provided in the REACH registration dossiers. Out of these, the following uses were considered to be particularly relevant for occupational exposure and therefore the focus of the assessment was laid on:

1. Manufacturing of diisocyanates,
2. Use in manufacture of PUs and PU composite materials,
3. Use in manufacture of foam,
4. Use in spray foam applications,
5. Use in coatings, and
6. Use in adhesives

Since the grouping of measurement data to industry groups and area groups as done by IFA is not based on use classifications as in the registration dossiers, a simple one-to-one translation/assignment of the MEGA data to the registered uses was not possible. The same holds true for all data provided by HSE and the publicly available data identified during the literature research. However, to allow a meaningful comparison of all identified data it was necessary to assign all data in a consistent way to the uses above. This was based on expert judgement in a way deemed most plausible. More details on the selection and grouping of the original data can be found in the restriction report (German CA, 2017).

Unless stated otherwise, the exposure levels shown in this paper are given in the same metrics as in the original sources. For most of the data, exposure values refer to the measured masses (concentrations) of the respective monomers. However, since the NCO unit is assumed to be the toxicologically relevant functional group, it is difficult to compare exposure data for the different species (MDI, TDI, and HDI) and, if applicable, their polyisocyanates as such (Bello *et al.*, 2004). For direct comparison of exposure values of different diisocyanates in the discussion part, the units were therefore converted into ‘total isocyanate group’ values (µg NCO m^−3^).

### Dermal exposure

In addition to inhalation exposure, there is an obligation to assess dermal exposures in the worker exposure scenarios under REACH. The dermal route is of particular relevance when assessing the overall exposure to diisocyanates, as the role of skin exposure to diisocyanates for development of respiratory sensitization and occupational asthma is discussed (Fent *et al.*, 2009; Tsui *et al.*, 2020). Cowie *et al.* conducted a comprehensive study on where diisocyanates were used in the UK and found that the airborne diisocyanate exposure were minimal in almost all of the investigated workplaces, but ‘there was nearly always potential for skin contact’ (Cowie *et al.*, 2005). Uncured or not fully cured PU products pose a source of skin exposure to diisocyanates (Bello *et al.*, 2007). This aspect is of particular importance since it was demonstrated that diisocyanates (e.g. from uncured resins) can deposit on and penetrate into the skin (Liljelind *et al.*, 2010). While airborne exposures could in principle be reduced by changing to less volatile diisocyanates (like MDI and/or prepolymers) for many uses, such substitutions do not necessarily reduce dermal exposure and there are ample opportunities for skin contact in workplaces. However, the assessment of dermal exposure at workplaces is often complicated by the irregular and random occurrence of skin exposure, such as spills, contact with contaminated surfaces or during clean-up (Bello *et al.*, 2007; Heederik *et al.*, 2012) and quantification of dermal exposure is particularly difficult. Measurement of dermal exposure, in general, is less established than air monitoring (Kasiotis *et al.*, 2020) and data on dermal exposure to diisocyanates in workplaces are scarce (Liu *et al.*, 2007). There are no standardized methods available for measuring dermal diisocyanate exposure (Lockey *et al.*, 2015). Sampling of dermal exposure to diisocyanates is challenging as the analytical methods are adaptations of the methods for airborne diisocyanates and rely on presence of unreacted NCO groups. Because NCOs (and especially mixtures of diisocyanates with polyols or amines) are highly reactive and they also react with moisture on or proteins of the skin, timing of the sampling is particularly critical (Redlich, 2010). For these reasons, dermal exposure to diisocyanates often is assessed indirectly by comparison of personal air samples with corresponding biomonitoring data (Cocker, 2011; Jones *et al.*, 2017). However, as measurement data on dermal exposure to diisocyanates are very limited, they will not be further discussed in this paper.

## Results

1. Manufacturing of diisocyanates

**Figure 1.**
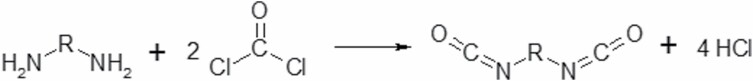
Phosgenation of a diamine for production of a diisocyanate.

The main process to produce diisocyanates is the phosgenation of corresponding diamines (see Fig. 1):

Owing to the dangerous properties of phosgene and isocyanates themselves, the production processes are carried out under containment in high integrity closed systems (Falcke *et al*., 2017). As long as the manufacturing processes run under normal operating conditions, occupational exposure to diisocyanates at this stage is generally considered to be low compared with the uses covering the application phases. This view is supported by occupational exposure data in the exposure scenarios for MDI and TDI published by ISOPA (2012a,b, 2014a,b,c). The exposure estimates of the diisocyanate species for manufacture of the diisocyanates as taken from the CSRs are in following ranges:

- MDI: 5.6–29 µg m^−3^;
- TDI: 5–32 µg m^−3^;
- HDI: 3–23.5 µg m^−3^.

These ranges are based on 90th percentiles of occupational hygiene measurement data and cover all contributing scenarios within the manufacturing scenarios.

2. Use of diisocyanates in manufacture of PUs and PU composite materials

**Figure 2.**
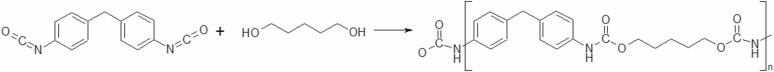
Reaction of MDI and 1,5-pentanediol.

Production of PU materials is the predominant use of diisocyanates and has the by far highest volume. To produce PUs the diisocyanates are reacted with macropolyols and/or other polynucleophiles and usually optional additives like catalysts, surfactants, stabilizers, flame retardants, and the like. The polyaddition reaction of isocyanates with the nucleophiles is highly exothermic. Depending on the reaction quantities and conditions, the temperature can increase considerably during the process. The chemical equation below exemplifies the general mechanism of the reaction by the example of MDI and 1,5-pentanediol (see Fig. 2). It is based on a simple single-phase PU, which is just one species of reaction products between diisocyanates and diols:

Usually the reaction is largely completed within seconds up to 30 min, whereby the isocyanate groups form urethane bonds with the polyol in the polymer backbone. However, the final curing and post-curing of PUs, where exposure to unreacted isocyanates is still possible may take up to 72 h. Occupational exposure often takes place on a regular basis in the production of PU materials and can be expected to be frequent. On the other hand, exposure control measures by means of technical controls/measures are often applied at such workplaces, so that exposure levels can be expected to be moderate.

MDI is the most used diisocyanate species for production of PU materials. Compared with MDI, TDI plays a subordinate role in the production of PU materials, except for the production of block foams, which are considered separately later in this paper. The use of and data on HDI in the manufacture of PUs on the other hand is very limited and therefore not taken into account for this use.

Table 1 provides an overview of the inhalation exposure levels to MDI and TDI in the manufacture of PU materials. The data are based on information given in the CSRs, by the Institute for Occupational Safety and Health of the German Social Accident Insurance (IFA, 2010), the Health and Safety Executive of the United Kingdom (HSE UK) (due to the confidential nature of the data provided, the following statement has to be included to this section: ‘The data is not representative of any industry partly due to bias in selection of the sites where data has been collected and is determined by HSE interest in specific substance or process. Most of the data was collected between 1986 and 1993 after which the rate of data collection reduced significantly. It should be noted that NEDB itself has an inherent bias, in that HSE Specialist Occupational Hygiene Inspectors as part of their enforcement duties obtained approximately 90% of the samples. Consequently, a tendency towards high levels of exposure would be expected, as companies with no perceived problems were generally not sampled. Even so, NEDB still contains many samples indicating low exposure (<25% of the appropriate occupational exposure limit), so the actual bias is not as large as would be expected. Whether or not NEDB should be considered as containing worst case data is debatable, but it cannot be regarded as being truly representative of occupational exposure in Great Britain given that it does not come from a random selection of workplaces and circumstances.’), and literature data on measured exposure levels of diisocyanate at workplaces in the PU and PU composite materials industry.

**Table 1.** Occupational inhalation exposure levels of diisocyanate species (µg m^−3^) for use of diisocyanates in manufacture of PUs and PU composite materials.

	CSRs 90th perc. range Long term/short term	IFA 90th perc. range (Mean) Long term	HSE Range Long term	Literature data Range (Mean/median)
MDI	2–38/3–76	<LoQ^a^–18.0 (Mean 2.3) (*N* = 559)	0.09–32.8 (*N* = 13)	• <0.03–3.3 (mean 0.7) (*N* = 131) [1]
				• 0.042–7.8 (med. 3.7) (*N* = 10) [2]
				• <1–7.2 (*N* = 70) [3]
				• <0.6–3.3 (*N* = 46) [4]
TDI	1–32/1–64	4.0–67.3 (Mean 1.3) (*N* = 293)	—	• 0.08–14.6 (med. 1.2–3.9) (*N* = 14) [2]

^*a*^LoQ was not further specified in IFA (2010) report; [1] Kääriä *et al.* (2001b); [2] Sennbro *et al.* (2004); [3] Creely *et al.* (2006); [4] Brzeźnicki and Bonczarowska (2015).

3. Use of diisocyanates in manufacture of foams

PU foams are generally divided by their elasticity into flexible, semi-flexible, and rigid foams. Foams are also the largest market for PUs, with flexible foams being the larger part. Both, MDI and TDI are used in the production of foams. High molecular polyols with two to six hydroxy functionalities yield flexible foams. When combined with low molecular polyols and/or amines, semi-flexible foams can be realized, while rigid foams are made of highly branched polyols with a relatively low molecular mass (Adam *et al.*, 2005). As very different manufacturing processes may be applied for different foams, generally a distinction is made between slab-stock foaming processes, foam moulding, and spray foaming, the latter of which is discussed separately (see below). Foam moulding can be seen as a special case in the manufacture of PU materials as described above, where the diisocyanate-containing component is mixed with the polyol and other components just before getting injected/transferred into moulds. As the reaction mostly takes place in the moulds this also allows closed systems. Slab-stock foaming on the other hand is a process where typically two (or more) polymer components are thoroughly combined in a mixing head and are then immediately dispensed onto conveyor belts. The polymerization takes place simultaneously to the foam formation of the slab-stock and usually just takes a few seconds but depending on the choice of the reacting agents can also last up to several minutes. While technical control measures to reduce exposures are usually applicable at the mixing unit and the following part of the conveyer belt (e.g. enclosure with enhanced exhaust ventilation) the end of the lines where curing of the slab-stock still takes place are open and exposure to residual diisocyanates is highly possible. After initial curing the slab-stock is cut and further processed. However, as the curing might have not completely finished, exposure to unreacted diisocyanates might be still possible at these stages (Cummings and Booth, 2002).

Due to the relatively low vapour pressure of MDI the ranges of inhalation exposure to MDI in manufacture of foam are usually low compared with TDI in the manufacture of foams. TDI is an important component in the production of flexible foams, mostly produced in slab-stock foaming processes. HDI is not considered to be relevant for the manufacture of foams.

Data from CSRs, IFA, HSE UK, and literature for inhalation exposures to MDI and TDI during the manufacture of foams are presented in Table 2. Some attention should be given to the values from the study by Tinnerberg and Mattsson (2008), where workplace measurement data from 13 Swedish industry plants were compared before and after installation of technical measures to improve the processes in order to reduce occupational exposures. After the modernization and improvements of the plants (mostly achieved through technical measures such as better enclosures, increased ventilation, and decreased reaction speed by reducing the amount of catalyst in the reaction mixture), the exposure levels were found to be around 80% lower compared with the levels before.

**Table 2.** Occupational inhalation exposure levels of diisocyanate species (µg m^−3^) for use of diisocyanates in manufacture of foams.

	CSRs 90th perc. range Long term/short term	IFA 90th perc. range (Mean) Long term	HSE Range Long term	Literature data Range (Mean/median)
MDI	6–29/12–58	<LoQ^a^–4.2 (Mean 1.7) (*N* = 1013)	0.03–0.17 (*N* = 3)	• <0.6 (*N* = 26) [4]
				• <0.6 (*N* = 20) [5]
TDI	1–32/1–64	<1.3–72.8 (Mean 4.7) (*N* = 110)	0.06–9.0 (*N* = 14) [Short term: 1.37–45.0 (*N* = 13)]	• <0.2–230 (*N* = 96) [1b]
				• 0.08–39.9 (med. 1.2–31.4) (*N* = 140) [2]
				• 0.2–58.8 (med. 4.0–9.8) (*N* = 26) [4]
				• 0.2–58.9 (mean 3.6–26.3) (*N* = 20) [5]
				• 46.5–73.6 (med. 62.9)^b^, 5.0–86.5 (med. 12.5)^c^ [6]
				• <7.2–17.4 (*N* = 26) [7]
				• 4.2–142 (mean 31.1) (*N* = 21) [8]
				• <0.71 (49 workers) [9]
				• 0.03–3.1 (5 workers) [10]

^*a*^LoQ was not further specified in IFA (2010) report.

^*b*^Before risk management measures (RMM) improvements.

^*c*^After RMM improvements; [1b]  Kääriä *et al.* (2001a); [2] Sennbro *et al.* (2004); [4] Brzeźnicki and Bonczarowska (2015); [5] Świerczyńska-Machura *et al.* (2015); [6] Tinnerberg and Mattsson (2008); [7] (Austin (2007); [8] Geens *et al.* (2012); [9] Gui *et al.* (2014); [10] Jones *et al.* (2017), levels given as total NCO (µg NCO m^−3^).

4. Use in spray foam applications

As spray foam applications are linked to particularly high exposure levels, compared with uses that take place in technically controlled environments (e.g. manufacture) or where only low mechanic energies are applied and therefore no or very low aerosol formation is to be expected (e.g. gluing), this use is considered as a special case.

Spray foams are typically two-component rigid foams, with one component being an isocyanate containing hardener (usually MDI based) and the other component being a polyol formulation (including catalysts, the blowing agent, and other additives such as flame retardants, surfactants, etc.). Depending on the formulation, open and closed cell foams can be realized. For application, the two components are pumped from separate containers into a spray gun, which also serves as the mixing unit. The reactivity of spray foam systems is usually very high so that the finished foam forms within seconds after spraying (Adam *et al.*, 2005). Spray foams are mainly used for insulation of buildings or industrial installations but can also serve as a speciality packing material for fragile items. This use is particularly challenging in terms of exposure reduction and risk management as aerosol formation during spraying is inevitable. In addition, spray foam installation is often carried out in dynamic workplaces (e.g. at construction sites) in which cases all equipment, including the technical measures (e.g. mobile enclosures) has to be mobile as well, which makes technical exposure reduction measures more challenging. When spray foams are applied in confined spaces (e.g. insulation of crawl spaces under basement, etc.), some technical measures like enclosures or exhaust ventilation might be not feasible at all or to a limited extend. Risk management and exposure reduction therefore largely depend on personal protective equipment (Allport *et al.*, 2003).

According to information from literature and the registration dossiers, the only diisocyanate used for spray foam applications is MDI. Table 3 summarizes the inhalation exposure levels to MDI during spray foam applications based on data from CSRs, IFA, HSE UK, and selected literature.

**Table 3.** Occupational inhalation exposure levels (µg m^−3^) for use of MDI in spray foam applications.

	CSRs 90th perc. range Long term/short term	IFA 90th perc. range (Mean) Long term	HSE Range Long term	Literature data Range (Mean/median)
MDI	6–29/11–58	<LoQ^a^ (Mean 1.9) (*N* = 33)	0.03–200 (*N* = 8)	• 0.07–2.47 (*N* = 36) [10]
				• 10–570 (*N* = 61) [11]
				• 70–2050 (*N* = 13) [12]
				• 11–591 (med. 54.8) (*N* = 94) [13]
				• <LoQ–770 [14]
				• <4.6–410 [15]
				• 30–90 (experimental set) [16]
				• 0.9–123.0 (GM 13.8) (*N* = 62) [17]

^*a*^LoQ was not further specified in IFA (2010) report; [10] Jones *et al.* (2017), levels given as total NCO (µg NCO m^−3^); [11] Crespo and Galán (1999); [12] Lesage *et al.* (2007); [13] Roberge *et al.* (2009); [14] RPS (2014); [15] Robert *et al.* (2014); [16] Puscasu *et al.* (2015); [17] Bello *et al.* (2019).

5. Use in coatings

Coatings are often applied to surfaces by spreading or by spraying, and as during these applications often aerosols are generated and/or splashes occur, it is therefore often linked to particularly high exposures in comparison to uses with no (or minimal) aerosol/droplet formation.

PU coatings can be one-component (one-pack) or two-component systems. One-pack paints containing free isocyanates are usually high molecular prepolymers of polyols with excess isocyanate groups that undergo a cross link reaction with atmospheric moisture. Two-component systems form the ‘conventional’ PU coatings and paints and are by far the most important systems (Adam *et al.*, 2005). The cross-linking constituents are polyisocyantes based on TDI, HDI, isophorone diisocyanate (IPDI), MDI, or 4,4'-methylenedicyclohexyl diisocyanate (HMDI). Solvent borne curing agent solutions have an isocyanate content of 5–16% (w/w), while solvent free may have up to 30% (w/w) isocyanate (Stoye *et al*., 2000). The other component of the paint contains polyols and/or polyamines as well as additives such as pigments, catalysts, and solvents. Both components are mixed immediately before application (preferably in an equimolar ratio). Due to their outstanding properties (especially high mechanical resistance, chemical resistance, and light and weather resistance) PU coatings are the systems of choice for protecting coatings like vehicle finishes and refinishes and in the building sector (floor coatings, anti-corrosion coatings, etc.). Aliphatic isocyanates (especially HDI) are important basic materials for protective and decorative coating systems, especially in vehicle body repair, where HDI-based spray paints are widely used.

All of the diisocyanate species covered in this assessment (MDI, TDI, and HDI) are used in coatings. Inhalation exposure to MDI as well as TDI during application of diisocyanates containing coatings is found to be relatively low compared with systems based on the more volatile HDI. Table 4 summarizes the inhalation exposure levels to MDI, TDI, and HDI for the use in coatings based on data from CSRs, IFA, HSE UK, and found literature relevant to the topic.

**Table 4.** Occupational inhalation exposure levels (µg m^−3^) for use of diisocyanates in coatings.

	CSRs 0th perc. range Long term/short term	IFA 90th perc. range (Mean) Long term	HSE Range Long term	Literature data Range (Mean/median)
MDI	6–29/11–58	<LoQ^a^–18.8 (Mean 2.4) (*N* = 685)	—	•0 .06–8.1 [10]
TDI	1–35/1–70	<1.3–6.0 (Mean 1.3) (*N* = 809)	—	—
HDI	110–430	<2.3–12.0 (Mean 2.3) (*N* = 1221)	0.35–208 (*N* = 15) [Short term: 0.82–245 000 (*N* = 47)]	• 421–423 [10]
				• med. 133–716 (*N* = 153) [18]
				• 0.02–57.6^b^ (med. 0.08–7.4) (*N* = 95) [19]
				• 0.003–179 (GM 3.2) (*N* = 88) [20]
				• 0.02–946.7^b^ (GM 87.2) [21]

^*a*^LoQ was not further specified in IFA (2010) report.

^*b*^Data are presented for monomeric HDI; [10] Jones *et al.* (2017), levels given as total NCO (µg NCO m^−3^); [18] Sparer *et al.* (2004); [19] Pronk *et al.* (2006a); [20] Fent *et al.* (2009); [21] Bello *et al.* (2020).

6. Use in adhesives

PU adhesives are used in a broad scope of applications and products ranging from extremely stable and weatherproof woodworking and construction glues to bonding automotive parts (e.g. windshields). The adhesives can be two-component or one-component systems, which themselves can be solvent-based, water-borne (aqueous dispersions), or solvent free (granulates, dry powders). They can be processed and/or cured at ambient temperatures or at elevated temperatures (from 50–80 to 180–200°C). With respect to potential exposure, it was shown that both the content of isocyanate monomers and the processing temperature have a significant impact on emissions (Cuno *et al.*, 2015).

Besides the broad spectrum of applications, there is only limited data available for the use of diisocyanates in adhesives. In fact, many of such systems, especially those that are used at room temperature, are often low emission glues.

Table 5 provides an overview of the inhalation exposure levels to MDI, TDI, and HDI in the use of adhesives as given in the CSRs, the MEGA evaluations by IFA, and as published by Brzeźnicki and Bonczarowska (2015).

**Table 5.** Occupational inhalation exposure levels (µg m^−3^) for use of diisocyanates in adhesives.

	CSRs 90th perc. range Long term/short term	IFA 90th perc. range (Mean) Long term	HSE Range Long term	Literature data Range
MDI	5–43/9–87	<LoQ^a^–6.5 (Mean 2.8) (*N* = 533)	—	<0.6–5.2 (*N* = 20) [4]
TDI	1–35/1–70	<1.3–48.2 (Mean 1.9) (*N* = 308)	—	—
HDI	—/—	<2.3 (*N* = 294)	—	0.8–1.0 (*N* = 20) [4]

^*a*^LoQ was not further specified in IFA (2010) report; [4] Brzeźnicki and Bonczarowska (2015).

### Biological monitoring

In addition to inhalation data some studies also provide biological monitoring data. Biological monitoring of diisocyanates is based on the analysis of isocyanate adducts with haemoglobin or albumin in the blood or the determination of corresponding diamines in urine or in plasma (Cocker, 2011; Świerczyńska-Machura *et al.*, 2015). The most common way for biomonitoring is using urine samples and looking for corresponding diamines of the diisocyanates, i.e. methylene diphenyl diamine (MDA) for MDI, toluene diamine (TDA) for TDI, and hexamethlyene diamine (HDA) for HDI. However, as the amines are not specific markers for diisocyanates, exposure to the corresponding diamines has to be ruled out since, otherwise, the results can be biased (Gries and Leng, 2013).

Table 6 provides summaries of the biological monitoring data for MDI in the manufacture of PUs and PU composite materials, for TDI in the manufacture of foam, and HDI in coatings.

**Table 6.** Overview of biological monitoring data of exposed workers.

Biomonitoring metabolite (µmol mol^−1^ creatinine) If not stated otherwise	Airmonitoring concentration (µg m^−3^)	Reference
*MDI in the manufacture of PUs and PU composite materials*		
0.015–1.38 (MDA in urine)	<0.03–3.3 (64% <0.03)	Kääriä *et al.* (2001b)
• 90th perc. 6.29 nmol l^−1^, median 1.34 (MDA in urine)		Sabbioni *et al.* (2007)
• 90th perc. 0.177 pmol g^−1^ Hb (haemoglobin adduct MDA)		
<LOD–12.64 (MDA in urine)	<1–7.2	Creely *et al.* (2006)
• 0.1–0.2 (MDA in urine, during working day)	—	Henriks-Eckerman *et al.* (2015)
• <0.1 (MDA in urine, day off)		
<LOD–12.64 (MDA in urine)	—	Robert *et al.* (2007)
• <LOD–14.1 (MDA in urine)	—	Gries and Leng (2013)
• <LOD–16.2 pmol g^−1^ (ABP-Val-Hyd in blood)		
• 0.5–8.4 µg l^−1^ (MDA in urine)	0.04–9.7	Tinnerberg *et al.* (2014)
• 0.4–19.4 µg l^−1^ (MDA in plasma)		
*TDI in the manufacture of foam*		
0.05–39 (total TDA in urine)	<0.2–230	Kääriä *et al.* (2001a)
<0.05–1.6 (total TDA in urine)	<3.5–8.4	Austin (2007)
Before RMM improvements:		Tinnerberg and Mattsson (2008)
• 2.9–27.2 µg l^−1^, median 7.0 (2,4-TDA in plasma)	46.5–73.6, median 62.9	
• 8.2–62.1 µg l^−1^, median 30.8 (2,6-TDA in plasma)		
After RMM improvements:		
• 0.5–2.0 µg l^−1^, median 1.0 (2,4-TDA in plasma)^b^	5.0–86.5, median 12.5	
• 2.0–11.8 µg l^−1^, median 4.0 (2,6-TDA in plasma)^b^		
• <LOD–3.9 (total TDA in urine)	0.2–58.9	Świerczyńska-Machura *et al.* (2015)
• <LOD–5.4 (total TDA in urine)	0.03–3.1^a^	Jones *et al.* (2017)
*HDI in coatings*		
• 1.9–146.2 (HDA in urine)	0.03–28.8 (HDI monomer)	Pronk *et al.* (2006b)
• <LOD–21.0 (HDA in urine)	0.003–179 (HDI monomer)	Gaines *et al.* (2010)
Before SHAD^b^:	—	Jones *et al.* (2013)
• 90th perc. 1.34 (HDA in urine)		
After SHAD:		
• 90th perc. 0.6 (HDA in urine)		
• <LOD–1.0 (total HDA in urine)	421–423^a^	Jones *et al.* (2017)

^*a*^Exposure levels given as total NCO (µg NCO m^−3^).

^*b*^Safety and Health Awareness Day.

As biological monitoring assesses the total burden of workers, it is not possible to distinguish between the exposure pathway and sources contributing to the burden. Diisocyanate metabolites can often be detected in biological monitoring samples even if the corresponding air monitoring measurements were below the limit of detection (Creely *et al.*, 2006). On the other hand, biological monitoring can be used to assess also behavioural aspects regarding the effectiveness of risk management measures like proper use of PPE and efficacy of training interventions (Jones *et al.*, 2013). However, recently the need for a harmonized approach for biological monitoring of isocyanates and to establish a baseline against which the effectiveness of the proposed restriction can be evaluated was highlighted (Scholten *et al.*, 2020).

### Other uses

As stated before, diisocyanates are used in a wide range of sectors and products, not all of which are covered by this assessment. It is clear that other diisocyanates like 1,5-naphthylene diisocyanate or IPDI can also have an impact on the workplace exposure (e.g. Tinnerberg *et al.*, 2014). Potentially high exposures can also arise in other uses than those described here [e.g. in foundry applications (Liljelind *et al.*, 2010)]. However, as information on those diisocyanates and/or uses was found to be too limited to allow further assessments of these exposure situations such aspects are not taken into the scope of this study.

## Discussion

**Figure 3.**
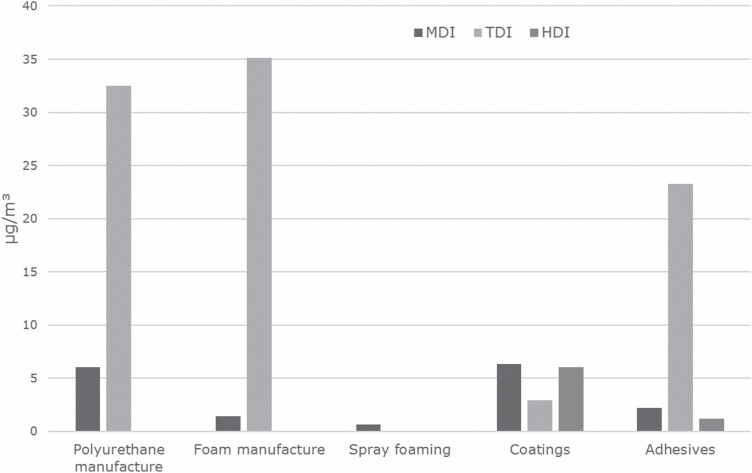
90th percentile values of air concentrations of MDI, TDI, and HDI for work area groups for selected uses from IFA reports (MEGA database; IFA, 2010, 2012, 2013), converted into total NCO units (µg NCO m^−3^).

The potential for occupational exposure to diisocyanates is determined by intrinsic substance properties (e.g. volatility) or to the processes involved in their handling. Volatility is one key determinant for the potential for inhalation exposure and related to the molecular size. Diisocyanates with a low molecular weight, i.e. TDI and HDI, have significant vapour pressures already at room temperature, which can lead to relatively high concentrations at the workplace (i.e. the respective diisocyanates can be detected and the concentrations are measurable, which is not the case e.g. for MDI). This trend can be also seen in the evaluations of the MEGA database for diisocyanates made by IFA as shown in Fig. 3 (IFA, 2010, 2012, 2013).

**Figure 4.**
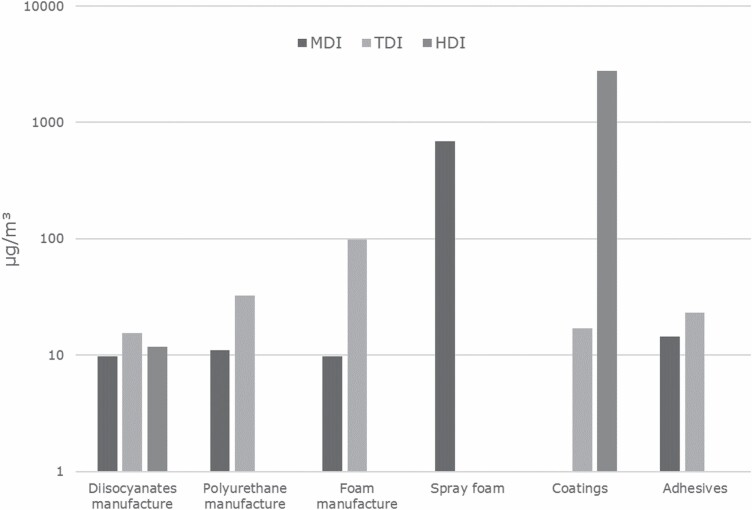
Air concentrations of MDI, TDI, and HDI for uses from all sources presented before, converted into total NCO units (µg NCO m^−3^) (note the logarithmic display of the exposure levels).

In addition, higher temperatures increase the vapour pressure thus the tendency of diisocyanates to become airborne is correspondingly higher in hot processes. In addition, handling diisocyanate-containing products at high temperatures can lead to thermal degradation, which can release the original monomeric diisocyanate and other low molecular isocyanates or fragments during thermal decomposition processes (Simon *et al.*, 1988; Delebecq *et al.*, 2013; Wang *et al.*, 2013). Very high exposures are found when a process is used where high levels of aerosols are formed (mostly spraying). Diisocyanate-based paints and varnishes are often used for spray painting; especially in vehicle body refinish HDI-based spray paints are ubiquitously frequently used and lead to significant occupational exposures. Spray foaming, especially when applied to greater surfaces (e. g. insulation of ceilings/walls) also leads to high aerosol release (Christensen *et al.*, 2014). Fig. 4 shows the found exposure levels for the different diisocyanates and sectors as presented before on a semi-logarithmic scale.

Many commercial products are not only based on diisocyanate monomers but can consist predominately of oligomers and/or prepolymers (e.g. in HDI-based coating systems). Nevertheless, the vast majority of available exposure data is based on the measurement of the respective diisocyanate monomer, whereas measurement of ‘total isocyanate group’ values (Bello *et al.*, 2004) has only recently become more common. It is understood that the overall risk of exposure to isocyanates is likely to be underestimated if these are not included. However, as written above, most of the available measurement data are for monomeric species, thus the focus in this paper is on these.

With regard to the dermal pathway, skin contact with products containing isocyanates (e.g. uncured PU foams, paint, or glue splashes) is reported to be a significant route of exposure (Austin, 2007) and there is almost always potential for dermal exposure when handling isocyanate containing formulations or reaction products thereof which are not fully cured (Cowie *et al.*, 2005). For example, Creely *et al.* found that urinary levels of isocyanate metabolites of workers with observable dermal exposure were over two times that of workers who did not have evident skin contact (Creely *et al.*, 2006). It was also observed that both dermal and inhalation exposures correlate significantly with urinary diisocyanate metabolite concentrations (Gaines *et al.*, 2010).

Grouping of the exposure data in a ranking order according to the reported bandwidths of inhalation exposure levels results in the following order, inhalation exposure levels to:

- HDI and its oligomers in coatings—from 0.003 up to 5566.3 µg m^−3^ (90th percentile), total range: 0.003–245 000 µg m^−3^
- MDI in spray foam applications—from limit of quantification (LoQ) up to 2050 µg m^−3^
- TDI in manufacture of foam—from LoQ up to 203 µg m^−3^
- TDI in manufacture of PUs and PU composite materials—from LoQ up to 67.3 µg m^−3^
- TDI in adhesives—from LoQ up to 48.2 µg m^−3^
- MDI in adhesives—from LoQ up to 43 µg m^−3^
- MDI in manufacture of PUs and PU composite materials—from LoQ up to 32.8 µg m^−3^
- TDI in coatings—from LoQ up to From LoQ up to 35 µg m^−3^
- MDI in manufacture of foam—from LoQ up to 29 µg m^−3^
- HDI in adhesives—from LoQ up to 1.0 µg m^−3^

The uses found to give rise to the highest inhalation exposure levels are HDI (and its oligomers) in coatings and MDI in spray foam applications. In both uses the diisocyanate resins are applied by spraying, confirming that high exposures are to be expected when diisocyanates are applied in high energy processes and aerosols are formed. Relatively high inhalation exposure levels are also found for some uses of TDI such as in the manufacture of foam as well as in the manufacture of PUs and PU composite materials and, in parts, for the use in adhesives. The exposure levels of MDI on the other hand are for all of these uses significantly lower. These findings are in line with the expectation that use of less volatile diisocyanates leads to lower inhalation exposure levels.

While for the majority of the discussed uses, most of the measured data were quite low (near or below the LoQ), it has to be stressed that measurement of airborne diisocyanates is technically challenging. The target compounds are usually highly reactive and some measurement methods are less sensitive to this, hence resulting in systematic underestimation of the actual exposure levels at workplaces (Streicher *et al.*, 2000, 2002; Bello *et al.*, 2004; Brandt *et al.*, 2013). Relatively high exposure levels can also occur in uses that appear to be well controlled at the first sight (e.g. TDI in adhesives). Such relatively high levels of inhalation exposure seem to occur in an unpredictable and unexplainable manner in all sectors and uses but could not be explained by analysis of the statistical data [see part B.9.9 of the Annex XV restriction dossier for diisocyanates (ECHA, 2017)]. The situation is further complicated by the fact, that different air sampling methods exist that measure differently (only monomers or total isocyanate mass concentrations, etc.), making comparison of measurement values between different studies more difficult (Bello *et al.*, 2004).

It should also be highlighted that isocyanate adducts can often be detected in biological monitoring samples even if the corresponding air monitoring measurements were below the limit of detection (e.g. Creely *et al.*, 2006). This might be explained by significant uncertainties in (i) the contribution of the respective exposure pathway to the total burden (inhalation vs. dermal) and (ii) uncertainties in the air monitoring data themselves as measurement of airborne diisocyanates and particularly of peak exposures is technically challenging and may underestimate actual exposure levels.

## Conclusion

The estimated number of annual incidences of diisocyanate-related occupational asthma in the EU is in the range from 2350 to 10150 cases. (ECHA. (2017) Annex XV restriction report—Diisocyanates—Part B. Helsinki, FI: ECHA.) The result of the exposure assessment(s) for diisocyanates at European workplaces lead to the conclusion that risks are not sufficiently controlled for a proportion of situations as shown above. Occupational exposure to diisocyanates is particularly relevant in:

- C.A.S.E. applications (Coatings, Adhesives, Sealants, Elastomers),
- production of PUs (e.g. slab-stock foam),
- handling of partly uncured PU products (e.g. cutting, demoulding, spray application of foam),
- when isocyanates/PUs are heated (e.g. hot lamination, foundry applications/casting forms).

With regard to the data for inhalation exposure it has to be kept in mind that measurement of airborne diisocyanates is technically challenging and may underestimate actual exposure levels. In addition, peak exposures to diisocyanates are particularly difficult to detect, which can also lead to an underestimation of exposure.

To address the risks of occupational asthma caused by diisocyanates, the German competent authority for REACH has proposed a restriction of products containing more than 0.1% by weight of diisocyanates (individually and in combination) under the EU’s REACH Regulation in 2016. The restriction was recently published in the EU Official Journal (European Commission, 2020) and will apply after a transitional period of 3 years from 24 August 2023. Diisocyanates are defined therein as ‘O=C=N–R–N=C=O, with R an aliphatic or aromatic hydrocarbon unit of unspecified length’. In the opinion of the authors this definition also applies to oligomers/prepolymers as long as they have two terminal NCO units. It is supposed that this will lead to increased substitution efforts to safer products, i.e. products containing less than 0.1% (w/w) of diisocyanates. However, a derogation from this ban can be made if industrial and professional users receive an obligatory standardized training on good working practices and risk management. Work training has been shown to be an effective measure to reduce occupational exposure to diisocyanates (Jones *et al.*, 2013) and the training required by the restriction aims to improve compliance and make working with diisocyanates safer.

## References

[CIT0001] Adam N, Avar G, Blankenheim H et al. (2005) Polyurethanes. In Ullmann’s Encyclopedia of Industrial Chemistry. Hoboken, NJ: Wiley-VCH.

[CIT0002] Allport DC, Gilbert DS, Outterside SM. (2003) MDI and TDI: safety, health and the environment: a source book and practical guide. Chichester, UK: John Wiley & Sons.

[CIT0003] Austin S . (2007) Biological monitoring of TDI-derived amines in polyurethane foam production. Occup Med (Lond); 57: 444–8. 17728315 10.1093/occmed/kqm085

[CIT0004] Bello D, Herrick CA, Smith TJ et al (2007) Skin exposure to isocyanates: reasons for concern. Environ Health Perspect; 115: 328–35. 17431479 10.1289/ehp.9557 PMC1849909

[CIT0005] Bello D, Woskie SR, Streicher RP et al (2004) Polyisocyanates in occupational environments: a critical review of exposure limits and metrics. Am J Ind Med; 46: 480–91. 15490474 10.1002/ajim.20076

[CIT0006] Bello A, Xue Y, Gore R et al (2019) Assessment and control of exposures to polymeric methylene diphenyl diisocyanate (pMDI) in spray polyurethane foam applicators. Int J Hyg Environ Health; 222: 804–15. 31076286 10.1016/j.ijheh.2019.04.014

[CIT0007] Bello A, Xue Y, Gore R et al (2020) Exposures and urinary biomonitoring of aliphatic isocyanates in construction metal structure coating. Int J Hyg Environ Health; 226: 113495. 32120250 10.1016/j.ijheh.2020.113495

[CIT0008] Brandt B, Assenmacher-Maiworm H, Hahn J. (2013) Messung und Beurteilung von Isocyanaten an Arbeitsplätzen unter Beachtung der TRGS 430. Gefahrst Reinhalt Luft; 73: 10.

[CIT0009] Brzeźnicki S, Bonczarowska M. (2015) Occupational exposure to selected isocyanates in Polish industry. Med Pr; 66: 291–301. 26325042 10.13075/mp.5893.00020

[CIT0010] Christensen F, Nilsson NH, Jeppesen CN et al (2014) Survey of certain isocyanates (MDI and TDI). Part of the LOUS-review. Copenhagen, DK: COWI A/S, Danish Technological Institute.

[CIT0011] Cocker J . (2011) Biological monitoring for isocyanates. Ann Occup Hyg; 55: 127–31. 21252056 10.1093/annhyg/meq083

[CIT0012] Cowie HAH, Graeme W, Creely KS et al (2005) An occupational hygiene assessment of the use and control of isocyanates in the UK. Edinburgh, UK: HSE Books.

[CIT0013] Creely KS, Hughson GW, Cocker J et al (2006) Assessing isocyanate exposures in polyurethane industry sectors using biological and air monitoring methods. Ann Occup Hyg; 50: 609–21. 16731584 10.1093/annhyg/mel024

[CIT0014] Crespo J, Galán J. (1999) Exposure to MDI during the process of insulating buildings with sprayed polyurethane foam. Ann Occup Hyg; 43: 415–9. 10518467

[CIT0015] Cummings BJ, Booth KS. (2002) Industrial hygiene sampling for airborne TDI in six flexible slabstock foam manufacturing facilities in the United States: a comparison of the short-term and long-term sampling data. Appl Occup Environ Hyg; 17: 863–71. 12495597 10.1080/10473220290107066

[CIT0016] Cuno E, Brandt B, Assenmacher-Maiworm H et al (2015) Emissionsverhalten von reaktiven Polyurethan-Schmelzkleb stoffen. Gefahrst Reinhalt Luft; 11–2: 457–64.

[CIT0017] Delebecq E, Pascault JP, Boutevin B et al (2013) On the versatility of urethane/urea bonds: reversibility, blocked isocyanate, and non-isocyanate polyurethane. Chem Rev; 113: 80–118. 23082894 10.1021/cr300195n

[CIT0019] ECHA . (2017) Annex XV restriction report—diisocyanates—part B. Helsinki, FI: ECHA.

[CIT0020] Engels HW, Pirkl HG, Albers R et al (2013) Polyurethanes: versatile materials and sustainable problem solvers for today’s challenges. Angew Chem Int Ed Engl; 52: 9422–41. 23893938 10.1002/anie.201302766

[CIT0021] European Commission. (2020) In Union OJE, editor. Commission Regulation (EU) 2020/1149 of 3 August 2020 amending Annex XVII to Regulation (EC) No 1907/2006 of the European Parliament and of the Council concerning the Registration, Evaluation, Authorisation and Restriction of Chemicals (REACH) as regards diisocyanates. Brussels, BE. Available at http://data.europa.eu/eli/reg/2020/1149/oj

[CIT0022a] Falcke H, Holbrook S, Clenahan I et al (2017) Toluene diisocyanate and methylene diphenyl diisocyanate. In Best available techniques (BAT) reference document for the production of large volume organic chemicals. Luxembourg: Publications Office of the European Union.

[CIT0022] Fent KW, Gaines LG, Thomasen JM et al (2009) Quantification and statistical modeling—part I: breathing-zone concentrations of monomeric and polymeric 1,6-hexamethylene diisocyanate. Ann Occup Hyg; 53: 677–89. 19622637 10.1093/annhyg/mep046 PMC2758668

[CIT0023a] Gabriel S, Koppisch D, Range D. (2010) The MGU–a monitoring system for the collection and documentation of valid workplace exposure data. Gefahrstoffe–Reinhalt Luft; 70: 43–49.

[CIT0023] Gaines LG, Fent KW, Flack SL et al (2010) Urine 1,6-hexamethylene diamine (HDA) levels among workers exposed to 1,6-hexamethylene diisocyanate (HDI). Ann Occup Hyg; 54: 678–91. 20530123 10.1093/annhyg/meq041 PMC2918490

[CIT0024] Geens T, Dugardin S, Schockaert A et al (2012) Air exposure assessment of TDI and biological monitoring of TDA in urine in workers in polyurethane foam industry. Occup Environ Med; 69: 93–8. 21725071 10.1136/oem.2011.064840

[CIT0025] German CA . (2017) Annex XV report; proposal for a restriction of diisocyanates—part B. Helsini, FI: ECHA.

[CIT0026] Gries W, Leng G. (2013) Analytical determination of specific 4,4′-methylene diphenyl diisocyanate hemoglobin adducts in human blood. Anal Bioanal Chem; 405: 7205–13. 23839327 10.1007/s00216-013-7171-z

[CIT0027] Gui W, Wisnewski AV, Neamtiu I et al (2014) Inception cohort study of workers exposed to toluene diisocyanate at a polyurethane foam factory: initial one-year follow-up. Am J Ind Med; 57: 1207–15. 25266741 10.1002/ajim.22385 PMC4198484

[CIT0028] Heederik D, Henneberger PK, Redlich CA; ERS Task Force on the Management of Work-related Asthma. (2012) Primary prevention: exposure reduction, skin exposure and respiratory protection. Eur Respir Rev; 21: 112–24. 22654083 10.1183/09059180.00005111 PMC9487304

[CIT0029] Henriks-Eckerman ML, Mäkelä EA, Laitinen J et al (2015) Role of dermal exposure in systemic intake of methylenediphenyl diisocyanate (MDI) among construction and boat building workers. Toxicol Lett; 232: 595–600. 25542146 10.1016/j.toxlet.2014.12.012

[CIT0030] IFA . (2010) MEGA evaluations for the preparation of REACH exposure scenarios for MDI and TDI (2000 to 2009) in Germany. Sankt Augustin, DE: Institute for Occupational Safety and Health of the German Social Accident Insurance (IFA).

[CIT0031] IFA . (2012) MEGA-Auswertungen zur Erstellung von REACH-Expositionsszenarien für Hexamethylen-1,6-diisocyanat (HDI). Sankt Augustin, DE: Institute for Occupational Safety and Health of the German Social Accident Insurance (IFA).

[CIT0032] IFA . (2013) MEGA-Auswertungen zur Erstellung von REACH-Expositionsszenarien für 2,4-Diisocyonattoloul (2,4-TDI) und 2,6-Diisocyonattoloul (2,6-TDI). Sankt Augustin, DE: Institute for Occupational Safety and Health of the German Social Accident Insurance (IFA).

[CIT0033] ISOPA . (2012a) MDI: final exposure scenarios in the e-SDS format [serial online]. Available at http://www.isopa.org/media/1663/esds-for-mdi-_30_apr_2012_-1.pdf. Accessed 29 September 2016.

[CIT0034] ISOPA . (2012b) MDI: final exposure scenarios in the e-SDS format [serial online]. Available at http://www.isopa.org/media/1663/esds-for-mdi-_30_apr_2012_-1.pdf. Accessed 29 September 2016.

[CIT0035] ISOPA . (2014a) Exposure scenarios—ISOPA communication in the supply chain on aromatic diisocyanates (MDI & TDI) & polyols [serial online]. Available at http://www.isopa.org/media/1627/isopa_use_descriptors_for_mdi-_tdi-_polyols_02_july_2014-1.pdf. Accessed 29 September 2016.

[CIT0036] ISOPA . (2014b) Socio-economic contribution of the polyurethane industry to growth and jobs in Europe. Brussels, BE: European Diisocyanate & Polyol Producers Association (ISOPA).

[CIT0037] ISOPA . (2014c) TDI: final exposure scenarios in the e-SDS format [serial online]. Available at http://www.isopa.org/media/1621/final-es-for-e_sds_tdi_update-july-2014-1.pdf. Accessed 29 September 2016.

[CIT0038] Jones K, Cocker J, Piney M. (2013) Isocyanate exposure control in motor vehicle paint spraying: evidence from biological monitoring. Ann Occup Hyg; 57: 200–9. 22986425 10.1093/annhyg/mes056

[CIT0039] Jones K, Johnson PD, Baldwin PEJ et al (2017) Exposure to diisocyanates and their corresponding diamines in seven different workplaces. Ann Work Expo Health; 61: 383–93. 28355438 10.1093/annweh/wxx006

[CIT0040] Kääriä K, Hirvonen A, Norppa H et al (2001a) Exposure to 2,4- and 2,6-toluene diisocyanate (TDI) during production of flexible foam: determination of airborne TDI and urinary 2,4- and 2,6-toluenediamine (TDA). Analyst; 126: 1025–31. 11478630 10.1039/b102022f

[CIT0041] Kääriä K, Hirvonen A, Norppa H et al (2001b) Exposure to 4,4′-methylenediphenyl diisocyanate (MDI) during moulding of rigid polyurethane foam: determination of airborne MDI and urinary 4,4′-methylenedianiline (MDA). Analyst; 126: 476–9. 11340982 10.1039/b009549o

[CIT0042] Kasiotis KM, Spaan S, Tsakirakis AN et al (2020) Comparison of measurement methods for dermal exposure to hazardous chemicals at the workplace: the SysDEA project. Ann Work Expo Health; 64: 55–70. 31785203 10.1093/annweh/wxz085

[CIT0043] Lesage J, Stanley J, Karoly WJ et al (2007) Airborne methylene diphenyl diisocyanate (MDI) concentrations associated with the application of polyurethane spray foam in residential construction. J Occup Environ Hyg; 4: 145–55. 17249149 10.1080/15459620601133779

[CIT0044] Liljelind I, Norberg C, Egelrud L et al (2010) Dermal and inhalation exposure to methylene bisphenyl isocyanate (MDI) in iron foundry workers. Ann Occup Hyg; 54: 31–40. 19783835 10.1093/annhyg/mep067

[CIT0045] Liu Y, Bello D, Sparer JA et al (2007) Skin exposure to aliphatic polyisocyanates in the auto body repair and refinishing industry: a qualitative assessment. Ann Occup Hyg; 51: 429–39. 17602207 10.1093/annhyg/mem021

[CIT0046] Lockey JE, Redlich CA, Streicher R et al (2015) Isocyanates and human health: multistakeholder information needs and research priorities. J Occup Environ Med; 57: 44–51. 25563538 10.1097/JOM.0000000000000278 PMC4286799

[CIT0047] Pronk A, Tielemans E, Skarping G et al (2006a) Inhalation exposure to isocyanates of car body repair shop workers and industrial spray painters. Ann Occup Hyg; 50: 1–14. 16126758 10.1093/annhyg/mei044

[CIT0048] Pronk A, Yu F, Vlaanderen J et al (2006b) Dermal, inhalation, and internal exposure to 1,6-HDI and its oligomers in car body repair shop workers and industrial spray painters. Occup Environ Med; 63: 624–31. 16728504 10.1136/oem.2005.023226 PMC2078164

[CIT0049] Puscasu S, Aubin S, Cloutier Y et al (2015) CIP10 optimization for 4,4-methylene diphenyl diisocyanate aerosol sampling and field comparison with impinger method. Ann Occup Hyg; 59: 347–57. 25452291 10.1093/annhyg/meu100

[CIT0050] Redlich CA . (2010) Skin exposure and asthma: is there a connection? Proc Am Thorac Soc; 7: 134–7. 20427586 10.1513/pats.201002-025RM PMC3266020

[CIT0051] Roberge B, Gravel R, Drolet D. (2009) 4,4′-Diphenylmethane diisocyanate (MDI)— safety practices and concentration during polyurethane foam spraying. Montréal, CA: Institut de recherche Robert-Sauvé en santé et en sécurité du travail (IRSST).

[CIT0052] Robert A, Ducos P, Francin JM et al (2007) Biological monitoring of workers exposed to 4,4′-methylenediphenyl diisocyanate (MDI) in 19 French polyurethane industries. Int Arch Occup Environ Health; 80: 412–22. 17061110 10.1007/s00420-006-0150-3

[CIT0052a] Robert W, Wood R, Andersen J.(2014) Spray polyurethane foam monitoring and re-occupancy of high pressure open cell applications to new residential constructions. In Polyurethanes Technical Conference, Dallas, TX. Available at https://polyurethane.americanchemistry.com/Products-Resources-and-Document-Library/SPF-Monitoring-and-Re-Occupancy-of-High-Pressure-Open-Cell-Applications-to-New-Residential-Constructions.pdf

[CIT0054] Sabbioni G, Wesp H, Lewalter J et al (2007) Determination of isocyanate biomarkers in construction site workers. Biomarkers; 12: 468–83. 17701746 10.1080/13547500701395636

[CIT0055] Scholten B, Kenny L, Duca R-C et al (2020) Biomonitoring for occupational exposure to diisocyanates: a systematic review. Ann Work Expo Health; 64: 569–85. 32313948 10.1093/annweh/wxaa038 PMC7328470

[CIT0056] Sennbro CJ, Lindh CH, Ostin A et al (2004) A survey of airborne isocyanate exposure in 13 Swedish polyurethane industries. Ann Occup Hyg; 48: 405–14. 15240337 10.1093/annhyg/meh034

[CIT0057] Simon J, Barla F, Kelemen-Haller A et al (1988) Thermal stability of polyurethanes. Chromatographia; 25: 99–106.

[CIT0057a] Smallenberg JA, van der Avert JM. (2014) Sprayed PUR foam emissions from crawl spaces. Unpublished report. RPS advise-en ingenieurbureau bv.

[CIT0058] Sparer J, Stowe MH, Bello D et al (2004) Isocyanate exposures in autobody shop work: the SPRAY study. J Occup Environ Hyg; 1: 570–81. 15559329 10.1080/15459620490485909

[CIT0058a] Stoye D, Funke W, Hoppe L et al (2000) Paints and Coatings. In Ullmann’s Encyclopedia of Industrial Chemistry. Hoboken, NJ: Wiley-VCH.

[CIT0059] Streicher RP, Reh CM, Key-Schwartz RJ et al (2000) Determination of airborne isocyanate exposure: considerations in method selection. AIHAJ; 61: 544–56. 10976685 10.1080/15298660008984567

[CIT0060] Streicher RP, Reh CM, Key-Schwartz R et al (2002) Selecting isocyanate sampling and analytical methods. Appl Occup Environ Hyg; 17: 157–62. 11871752 10.1080/104732202753438234

[CIT0061] Świerczyńska-Machura D, Brzeźnicki S, Nowakowska-Świrta E et al (2015) Occupational exposure to diisocyanates in polyurethane foam factory workers. Int J Occup Med Environ Health; 28: 985–98. 26294200 10.13075/ijomeh.1896.00284

[CIT0062] Tinnerberg H, Broberg K, Lindh CH et al (2014) Biomarkers of exposure in Monday morning urine samples as a long-term measure of exposure to aromatic diisocyanates. Int Arch Occup Environ Health; 87: 365–72. 23558852 10.1007/s00420-013-0872-y

[CIT0063] Tinnerberg H, Mattsson C. (2008) Usage of air monitoring and biomarkers of isocyanate exposure to assess the effect of a control intervention. Ann Occup Hyg; 52: 187–94. 18344533 10.1093/annhyg/men006

[CIT0064] Tsui HC, Ronsmans S, De Sadeleer LJ et al (2020) Skin exposure contributes to chemical-induced asthma: what is the evidence? A systematic review of animal models. Allergy Asthma Immunol Res; 12: 579–98. 32400126 10.4168/aair.2020.12.4.579 PMC7224990

[CIT0065] Wang H, Wang Q-S, He J-J, Mao Z-l, Sun J-H. (2013) Study on the pyrolytic behaviors and kinetics of rigid polyurethane foams. Procedia Eng; 52: 377–85.

