# Correction to: Occupational Exposure to Diisocyanates in the European Union

**DOI:** 10.1093/annweh/wxad027

**Published:** 2023-05-20

This is a correction to: Dag Rother, Urs Schlüter, Occupational Exposure to Diisocyanates in the European Union, Annals of Work Exposures and Health, Volume 65, Issue 8, October 2021, Pages 893–907, https://doi.org/10.1093/annweh/wxab021

In the originally published version of this manuscript, the estimated number of annual cases of diisocyanate-related occupational asthma given in the following sentence in the Conclusion section was incorrect:

‘The estimated number of annual incidences of diisocyanate-related occupational asthma in the EU is in the range from 2350 to 7269 cases.’

The sentence should instead read as follows:

‘The estimated number of annual incidences of diisocyanate-related occupational asthma in the EU is in the range from 2350 to 10150 cases. (ECHA. (2017) Annex XV restriction report—Diisocyanates—Part B. Helsinki, FI: ECHA.).’

These details have been corrected only in this correction notice to preserve the published version of record.

